# Utilization of Current Diagnostic Indicators to Characterize Pediatric Undernutrition among US Children

**DOI:** 10.3390/nu12051409

**Published:** 2020-05-14

**Authors:** Alyssa Archdeacon Price, Jennifer A. Williams, Holly Estes Doetsch, Colleen K. Spees, Christopher A. Taylor

**Affiliations:** 1 Dayton Children’s Hospital, Dayton, OH 45404, USA; pricea1@childrensdayton.org; 2 Abbott Nutrition, Columbus, OH 43219, USA; jennifer.williams@abbott.com; 3 The Ohio State University, Columbus, OH 43210, USA; Holly.Estes@osumc.edu (H.E.D.); spees.11@osu.edu (C.K.S.)

**Keywords:** undernutrition, malnutrition, pediatric, anthropometrics, *z*-scores

## Abstract

The purpose of this study was to estimate the prevalence of pediatric undernutrition in the US general population using the Academy of Nutrition and Dietetics/American Society for Parenteral and Enteral Nutrition consensus statement on identification of pediatric malnutrition (undernutrition). National Health and Nutrition Examination Survey (NHANES) data for years 2005–2014 was analyzed for children ages 1–13 years (*n* = 13,950) with valid anthropometric data. The prevalence of undernutrition was assessed through *z*-scores for weight-for-height, body mass index (BMI)-for-age, height-for-age, and mid-upper-arm circumference-for-age generated from the 2000 Centers for Disease Control and Prevention growth charts. Children were stratified into: no undernutrition, mild undernutrition, and moderate or severe undernutrition. Descriptive statistics were used to identify the prevalence of undernutrition. Differences in *Z*-scores across growth chart metrics were compared across undernutrition categories using analysis of variance. The total prevalence of pediatric undernutrition in this sample was 0.4% (severe undernutrition), 2.0% (moderate undernutrition), and 10.9% (mild undernutrition) for all ages. *Z*-scores differed significantly across all levels of undernutrition for all anthropometrics, showing poorer mean growth metrics in those with undernutrition. Pediatric undernutrition is a prevalent condition that transcends the prior focus on <5th percentile of growth curves and impacts children across different demographic categories.

## 1. Introduction

Malnutrition has been used synonymously to indicate a state of over- or under-nutrition, evidenced by an excess or deficiency in essential nutrients; however, when evaluating growth concerns in children related to insufficient nutritional status, malnutrition is defined through the lens of undernutrition. Anthropometric measures are the traditional method of growth assessment in pediatrics because they can be indicative of a child’s current and historical nutritional status [1]. In cases of acute undernutrition, weight may be impacted; however, if undernourishment becomes chronic, the child may have stunted height as well [1,2]. Historically, undernutrition has most commonly been described as a failure to thrive, a condition for which diagnostic criteria may vary by practitioner, with inconsistent nutrition therapy recommendations [2,3,4,5,6]. Undernutrition is associated with many long-term developmental and functional outcomes [7,8,9,10]. Growth failure at 24 m of age has been associated longitudinally with lower reading and intelligence scores, higher poverty rates, and lower levels of education attained [7,8,9,10]. Without proper intake of nutrients, children can be at risk for significant immune deficiencies, compromised cognitive function, behavioral problems, stunting, and muscle loss, especially if undernutrition is chronic in duration [8,9,10]. The World Health Organization (WHO) growth reference data indicates that children < 5 years are capable of similar growth potential with optimal nutrition [11,12].

Prior estimates of the prevalence and demographic characteristics of pediatric undernutrition and failure to thrive exist; however, these estimates are often based on hospitalized patients or may be confounded by disparities in screening practices [13,14,15]. Thus, these estimates may not represent the general population cared for in pediatric primary care settings. Further, growth faltering has been diagnosed using an expansive array of criteria and metrics of assessment that primarily centered on those who fell below the 5th percentile on pediatric growth curves [2,16,17,18]. To create a unified screening and diagnostic criteria for undernourished children, the Academy of Nutrition and Dietetics (AND) and the American Society for Parenteral and Enteral Nutrition (ASPEN) established a consensus statement on the identification of pediatric malnutrition (undernutrition) [13]. This statement provides recommended anthropometric indicators for assessment and diagnosis of the severity of undernutrition. Therefore, to promote support for optimal growth and development in US children, a broader assessment of the prevalence of undernutrition is needed.

With universal criteria established for the identification of pediatric undernutrition, there is an opportunity to better screen children and promote early nutrition intervention strategies to optimize growth and development. Therefore, the purpose of this analysis was to identify the prevalence of pediatric undernutrition in the general US population and identify the presence across various demographic categories. This information may serve as the foundation for the development of targeted interventions for improving nutrition status among specific subgroups of children. 

## 2. Materials and Methods 

### 2.1. Study Population

This analysis includes biennial data from the 2005–2014 years of the National Health and Nutrition Examination Survey (NHANES) for children aged 1–13 years old (*n* = 13,950) with complete and reliable anthropometric measures. NHANES is a multi-staged, stratified sample of the non-institutionalized US population. Difficult to reach populations, such as those from low-income households, racial and ethnic minorities, and young children, were oversampled to promote suitable representation in the data. Data were collected in 2-year cycles, and the data from the five cycles were combined into a single sample for this analysis. Consent and demographic data for NHANES were obtained during an in-home interview and a physical examination was conducted during the Mobile Examination Center visit. The Centers for Disease Control and Prevention (CDC) Institutional Review Board reviewed and approved data collection protocols.

### 2.2. Anthropometric Data

Publicly available data were extracted from the National Center for Health Statistics website, imported in Statistical Package for the Social Sciences (SPSS, version 24, IBM, New York, NY, USA) for recoding. Sex, age (months at the time of physical examination), height, weight, length, and mid-upper arm circumference (MUAC) were processed through EpiInfo (version 3.5.4, CDC, Atlanta, GA, USA) to generate *z*-scores based on the 2000 CDC growth charts for the growth metrics used to identify the prevalence of undernutrition using a single data point [13]. Recumbent length was assessed for children between the ages of birth through the first 47 months of age, and standing height was measured for any participant over the age of two years. If participants had measures for both recumbent length and standing height available, height-for-age *z*-scores were utilized for these analyses. MUAC was assessed on the right arm at the midpoint between the acromion process and olecranon process, and then compared to other children of the same age and sex between the ages of 6 and 59 months. Body mass index (BMI)-for-age was utilized for children over the age of 2 years.

### 2.3. Data Analysis

Pediatric malnutrition (undernutrition) was categorized based on *z*-scores that define the severity of undernutrition from the AND/ASPEN Pediatric Malnutrition Consensus Statement shown in Table 1 [13]. Criteria utilized to assess undernutrition status from the consensus statement include weight-for-height/length-for-age, BMI-for-age, height/length-for-age, and MUAC-for-age. Classification of pediatric undernutrition was established based on the most severe anthropometric *z*-score category per individual child. Those children classified with anthropometric criteria indicating moderate or severe undernutrition were combined in these analyses to generate more appropriate sample sizes for comparative analysis. 

Children were stratified into four age groups for analyses: 1 year old, 2–5 years, 6–8 years, and 9–13 years. Data were weighted using SPSS Complex Samples (Version 24.0) to generate nationally representative estimates, while also producing appropriate sample-based standard errors for statistical analysis. Descriptive statistics were utilized to assess the prevalence of undernutrition status by age group, race/ethnicity (non-Hispanic white, non-Hispanic black, Hispanic/Latino), and income category (<100%, 100%–185%, >185%–300%, >300% of the federal poverty level), in addition to the frequency categorizations by anthropometric markers used to diagnose undernutrition. Mean *z*-scores of anthropometric measures were compared using analysis of covariance across the levels of undernutrition, controlled for sex, race/ethnicity, and income as a percent of the federal poverty rate.

## 3. Results

Analysis was conducted using data from children 1 year old (*n* = 1428), 2–5 years (*n* = 4599), 6–8 years (*n* = 3087), and 9–13 years (*n* = 4836), with sample sizes for demographic and personal characteristics provided in Table 2. Table 3 presents the prevalence for the markers of undernutrition and the overall classifications of undernutrition by age category. The total prevalence of pediatric undernutrition in this sample was 0.4% (severe undernutrition), 2.0% (moderate undernutrition), and 10.9% (mild undernutrition) for all ages. Mild undernutrition was the most common subcategory of undernutrition, affecting 8%–21.6% across the age categories. Prevalence of undernutrition were lower in older age groups, but still affected about 10% of children aged 9–13 years old. MUAC was the most prevalent risk factor in those 5 years and younger, followed by weight-for-height-for-age. BMI-for-age was the predominant marker in children 6 years and older.

The undernutrition data was also characterized according to specific demographic characteristics—age, sex, race/ethnicity, and income-to-poverty ratio (Table 4). Prevalence of undernutrition was similarly prevalent across the various sociodemographic characteristics. Mild and moderate/severe undernutrition was most prevalent in non-Hispanic white children in the 1-year-old group, but highest in non-Hispanic black children for the 2–5-year-olds. For the 6–8-year-old age category, the prevalence of undernutrition was notably higher for the “other or multiracial” race/ethnicity group than other groups, at approximately 14%. Mild undernutrition was most prevalent in the 185%–300% or the >300% federal poverty rate income levels across all age groups. The prevalence of undernutrition was similar by sex. 

To assess the differences in growth metrics across levels of undernutrition, mean *z*-scores were compared for all markers of growth (Table 5). Regardless of the metric used to classify undernutrition, there were significant differences in mean *z*-scores across all growth metrics across each of the age categories (*p* < 0.001). Only children in the no indicators of undernutrition category had positive mean *z*-scores. Whereas, children who met at least one criterion for undernutrition had average *z*-scores of less than zero across all applicable measures, and children with at least one criterion for mild undernutrition had average *z*-scores of less than or equal to –1 for most anthropometric measures.

## 4. Discussion

Although there is a strong national focus on the prevalence of obesity in the US, the prevalence of undernutrition is another public health condition that should not be ignored [19]. In a nationally representative sample, these analyses showed the prevalence of undernutrition among differing demographics in the non-institutionalized pediatric population of the US. Prior data with varied definitions indicated that there is a prevalence of malnutrition/undernutrition between 5%–10% in primary care settings and between 3%–5% in hospital admissions [20,21]. Yet, inconsistencies in the practices used to determine undernutrition create challenges for comparing the prevalence.

Previous analyses have found the presence of pediatric undernutrition in the United States to be more prevalent in females, non-white racial/ethnic groups, and those uninsured or those supported by federal healthcare programs [14]. Data from the present study noted little difference by sex, and undernutrition was noted across all race/ethnicity and income categories. The prevalence of undernutrition across all age categories, as well as throughout socioeconomic factors, provides evidence for the need to promote interventions addressing poor growth at all stages of pediatric development, including consistent and frequent follow-ups that document growth velocity for children in the mild to moderate range of undernutrition. These data report that a considerable proportion of children meet a criterion of undernutrition up through age 13 years, which is counter to a conception that children with mild early growth limitations will “grow out of it” as they age [7,8,9,10,22,23]. Future efforts will need to explore longitudinal changes in growth to assess the precision of a malnutrition diagnosis, as well as necessary factors linked to appropriate growth and development catch up.

The consistent prevalence of pediatric undernutrition across sociodemographic categories found in this analysis elevates the importance of utilizing a simple and specific screening tool to diagnose pediatric undernutrition in the outpatient population. In addition to tracking anthropometrics over time, including growth velocity, registered dietitian nutritionists should employ strategies of nutrition-focused physical examination to assess contributing factors, and also identify clinical signs of undernutrition, which may include lethargy, irritability or change in mood, muscle and fat depletion, signs of micronutrient deficiencies, and recurrent illness or infections [24,25]. A diet record, assessment of any underlying disease, and family history to rule out constitutional growth delays or familial short stature can also help round out the clinical picture [26,27].

While the emphasis of undernutrition has been focused on underserved populations, assessment of growth data from a national sample illustrates that undernutrition transcends sociodemographic characteristics. All levels of undernutrition were evident in children above income thresholds for food assistance programs, across all ages, with the highest prevalence of mild undernutrition in those over 185% of the federal poverty rate. Assistance programs, such as The Special Supplemental Nutrition Program for Women, Infants, and Children (WIC), Supplemental Nutrition Assistance Program (SNAP), and the National School Lunch Program (NSLP), set standards and provide nutrition assistance to families that meet income eligibility requirements. Older children and those from higher income households would be less likely to meet requirements for these programs. With the overall poor dietary habits of US children, those within families above the threshold for government assistance may be at a higher probability for undernutrition. 

The American Academy of Pediatrics (AAP) recently issued a call to action on food security for children. This mandate may help improve screening practices so that pediatricians may better assess if children are receiving the necessary nutrition for growth. The AAP has noted that households of all incomes may be impacted by food insecurity, which can lead to undernutrition for those in the household. Strategies for alleviating this risk of undernutrition will likely require approaches to reach a broader net of children beyond the underserved. Since children from all socioeconomic levels, race/ethnicities, ages, and sexes are affected by undernutrition, all stakeholders, chiefly parents, schools, and pediatricians, should have access to appropriate nutrition education [28,29,30,31].

Prior to the recent pediatric malnutrition consensus statement, growth faltering may have been diagnosed by any of the following means: Gomez Criteria, Waterlow criteria, body mass-for-chronological age less than the 5th percentile, length for chronological age less than the 5th percentile, conditional weight gain equal to the lowest 5% adjusted for regression towards the mean, from birth until weight within the given age group, or, weight deceleration crossing over two major centile lines from birth until weight within the given age group [2,16,17,18]. Because of the wide range of assessment strategies previously utilized, identification of undernutrition has been extremely dependent on the specific criterion utilized. *Z*-scores have been identified as the most appropriate indicator of undernutrition in pediatrics and enable comparison to mean values for age and sex, along with evaluation of extreme values [32]. If a child’s *z*-score for an anthropometric measurement is below –1, previously used undernutrition criteria would have considered this an indication of faltering growth [8]. The mean negative *z*-scores for children in the undernutrition category for each anthropometric indicator assessed in this analysis provides validity to the definition, with differences in all growth measures at varying levels of undernutrition. Additionally, the results here indicate the need for continued validation and long-term functional outcome research to support the current AND/ASPEN consensus statement and provide guidance for practitioners in the US [13]. Identification and early intervention for undernutrition in pediatric patients is crucial, for these patients are undergoing rapid growth and development and may become undernourished more rapidly than adults [24]. Young children are at risk for irreversible outcomes, such as stunting and cognitive impairment, if energy and essential nutrients are not available during critical periods of development [24]. 

Previously, weight-for-height/length *z*-scores have been identified as the gold standard for identifying acute undernutrition in pediatrics [33]. MUAC has often been utilized as a proxy indicator for undernutrition, and, in these results, MUAC *z*-scores presented as the most prevalent risk factor in children under age 5 years. Because MUAC is a specific and powerful indicator of mortality, it is recommended that it be included in standard screening practice protocols for younger children at a minimum [29,30,31]. It has been suggested that both weight-for-height/length and MUAC-for-age should be used together to identify a child with undernutrition, and the results in this paper support their combined usage for identification of undernutrition in clinical practice [34,35]. 

While this data showing undernutrition in a national sample of free-living children is a strength, inherent limitations of the data must be considered. In these analyses, assessment of growth was from a cross-sectional sample at a single time point versus longitudinal growth data collection. As a result, the diagnostic criteria for undernutrition diagnosis from a single data point were used [16]. A single anthropometric measure will not demonstrate temporal changes in growth, and growth would be more optimally assessed longitudinally for diagnosis. However, this surveillance system uses established protocols to maintain accurate validity and reliability of anthropometric data collected, and technicians are continuously trained and monitored. The relatively small number of children presenting with undernutrition required the combination of multiple data collection cycles for stability of national estimates but limits the statistical power for subgroup analyses. Also, sexual maturation is not available in the data to account for anthropometrics in the older children.

The driver for the consensus statement was to create a mechanism to assess broader clinical endpoints that are related to health outcomes in child development. With limited data availability on the national prevalence and scope of undernutrition with these new criteria, these data provide a foundation for the need for advanced screening and assessment in US children to promote support of optimal growth and development. Future efforts may explore the temporal trends that may account for the variability and factors related to differences in growth outcomes over time. Overall, the analysis provides a snapshot of the prevalence of undernutrition among children in the United States. These data support a greater focus on growth assessment across all children, beyond those typically assumed to be at high risk, to promote optimal outcomes in growth and development.

## Figures and Tables

**Table 1:** Primary indicators of the severity of undernutrition-based *z*-scores from a single data point (modified from Becker et al. [13]).

Growth Indicators	Mild Undernutrition:	Moderate Undernutrition:	Severe Undernutrition:
Weight-for-height-for-age ^a^	−1 to −1.9 *z* score	−2 to −2.9 *z* score	≤ −3 *z* score
BMI-for-age ^b^	−1 to −1.9 *z* score	−2 to −2.9 *z* score	≤ −3 *z* score
Length/Height-for-age	No data	No data	≤ −3 *z* score
MUAC-for-age ^c^	−1 to −1.9 *z* score	−2 to −2.9 *z* score	≤ −3 *z* score

^a^ For children 1 year of age, weight-for-length-for-age was used for assessment. ^b^ BMI, body mass index. ^c^ MUAC, mid upper ar circumference.

**Table 2:** Unweighted sample sizes of demographic and undernutrition characteristics by age categories of US children 1–13 years of age (*n* = 13,950).

Characteristic	Category	1 Year (*n* = 1428)	2–5 Years (*n* = 4599)	6–8 Years (*n* = 3087)	9–13 Years (*n* = 4836)	Total (*n* = 13,950)
Sex	Male	747	2369	1602	2418	7136
	Female	681	2230	1485	2418	6814
Race/Ethnicity	Mexican American	409	1197	789	1291	3686
	Other Hispanic	161	468	309	457	1395
	Non-Hispanic White	424	1311	857	1298	3890
	Non-Hispanic Black	307	1132	805	1307	3551
	Other or Multiracial	127	491	327	483	1428
Income Category	<100% federal poverty rate	521	1663	996	1508	4688
	100%–185% poverty rate	308	1048	736	1075	3167
	>185%–300% poverty rate	207	629	439	768	2043
	>300% poverty rate	298	923	731	1174	3126
Survey Cycle	2005–2006	353	986	554	1104	2997
	2007–2008	296	888	608	885	2677
	2009–2010	318	939	597	931	2785
	2011–2012	220	905	654	938	2717
	2013–2014	241	881	674	978	2774
Undernutrition Category	No indicators	1087	3739	2785	4398	12,009
	Mild	286	725	242	344	1597
	Moderate/Severe	55	135	60	94	344

**Table 3:** Nationally-representative proportion of US children presenting with undernutrition criteria identified by growth *z*-scores across age groups and undernutrition prevalence according to age groups from National Health and Nutrition Examination Survey (NHANES) 2005–2014 data (*n* = 13,950).

Growth Measure	Level	1 Year	2–5 Years	6–8 Years	9–13 Years
Weight-for-height *z*-score ^a, b^	No Indicator	87.7%	87.3%	27.1%	0.1%
	Mild	9.4%	9.1%	3.0%	0.0%
	Moderate	1.9%	1.3%	0.7%	0.0%
	Severe	0.5%	0.2%	0.2%	0.0%
	Missing data	0.6%	2.2%	68.9%	99.8%
BMI-for-age ^c^ *z*-score	No Indicator		86.9%	90.3%	89.8%
	Mild		8.0%	7.9%	7.9%
	Moderate		1.6%	1.6%	2.0%
	Severe		0.1%	0.3%	0.3%
	Missing data	100%	3.3%	0.0%	0.0%
Height-for-age *z*-score ^a^	No Indicator	99.2%	99.4%	99.7%	100.0%
	Mild	0.0%	0.0%	0.0%	0.0%
	Moderate	0.0%	0.0%	0.0%	0.0%
	Severe	0.2%	0.2%	0.3%	0.0%
	Missing data	0.5%	0.4%	0.0%	0.0%
MUAC-for-age *z*-score ^d^	No Indicator	73.8%	61.7%		
	Mild	19.5%	9.9%		
	Moderate	2.4%	0.4%		
	Severe	0.1%	0.0%		
	Missing data	4.2%	28.0%	100%	100%
Pediatric Undernutrition Prevalence	No Indicators	74.8%	83.1%	89.8%	89.8%
	Mild	21.0%	14.5%	8.0%	7.9%
	Moderate	3.4%	2.1%	1.5%	2.0%
	Severe	0.7%	0.4%	0.7%	0.3%

Data are weighted to generate a nationally-representative sample. ^a^ Weight-for-length and length-for-age z-scores were used for 1-year-olds. ^b^ Weight-for-height *z*-scores were not presented for children with heights exceeding the growth chart maximum height. ^c^ BMI-for-age *z*-scores were not available for 1-year-olds. ^d^ MUAC-for-age *z*-scores were not assessed above age 5 years.

**Table 4:** Prevalence of pediatric undernutrition by sociodemographic characteristics and age categories in US children from NHANES 2005–2014 data (*n* = 13,950).

Age		Personal Characteristics	Mild Undernutrition	Moderate/Severe Undernutrition
1 Year	Sex	Male	21.7% (18.3%, 25.6%)	4.2% (2.7%, 6.6%)
		Female	20.3% (16.6%, 24.4%)	4.1% (2.6%, 6.4%)
	Race/ethnicity	Mexican American	17.2% (13.1%, 22.3%)	4.2% (2.6%, 6.7%)
		Other Hispanic	24.2% (17.3%, 32.6%)	2.7% (1.1%, 6.7%)
		Non-Hispanic White	22.5% (18.7%, 26.8%)	4.3% (2.6%, 7.0%)
		Non-Hispanic Black	20.0% (16.2%, 24.4%)	2.8% (1.3%, 5.8%)
		Other or Multiracial	18.1% (10.9%, 28.3%)	6.7% (2.7%, 15.8%)
	Household income	<100% federal poverty rate	17.6% (13.8%, 22.2%)	3.4% (2.2%, 5.3%)
		100%–185% poverty rate	21.4% (16.5%, 27.2%)	3.7% (2.1%, 6.5%)
		>185%–300% poverty rate	18.4% (13.4%, 24.7%)	7.2% (3.9%, 12.9%)
		>300% poverty rate	25.1% (20.1%, 30.9%)	3.4% (1.7%, 6.5%)
2–5 Years	Sex	Male	15.9% (14.1%, 18.0%)	2.0% (1.5%, 2.8%)
		Female	13.0% (11.2%, 15.1%)	2.9% (2.1%, 4.0%)
	Race/ethnicity	Mexican American	15.3% (13.2%, 17.8%)	2.6% (1.8%, 3.8%)
		Other Hispanic	10.9% (8.1%, 14.6%)	0.7% (0.2%, 1.9%)
		Non-Hispanic White	13.6% (11.7%, 15.9%)	1.8% (1.1%, 2.9%)
		Non-Hispanic Black	17.3% (14.9%, 20.0%)	3.8% (2.7%, 5.2%)
		Other or Multiracial	16.4% (12.2%, 21.8%)	5.4% (3.5%, 8.2%)
	Household income	<100% federal poverty rate	13.5% (11.8%, 15.3%)	3.0% (2.1%, 4.3%)
		100%–185% poverty rate	14.2% (11.6%, 17.3%)	2.1% (1.4%, 3.3%)
		>185%–300% poverty rate	16.7% (12.8%, 21.4%)	2.1% (1.1%, 3.8%)
		>300% poverty rate	14.3% (11.9%, 17.1%)	2.1% (1.3%, 3.5%)
6–8 Years	Sex	Male	7.3% (5.6%, 9.5%)	2.7% (1.9%, 3.9%)
		Female	8.8% (7.1%, 10.8%)	1.5% (0.9%, 2.5%)
	Race/ethnicity	Mexican American	5.1% (3.6%, 7.1%)	2.1% (1.4%, 3.3%)
		Other Hispanic	6.4% (4.3%, 9.5%)	1.9% (0.9%, 4.3%)
		Non-Hispanic White	8.3% (6.4%, 10.7%)	2.4% (1.4%, 3.9%)
		Non-Hispanic Black	7.2% (5.6%, 9.3%)	1.1% (0.5%, 2.2%)
		Other or Multiracial	14.0% (10.0%, 19.3%)	2.6% (1.3%, 5.1%)
	Household income	<100% federal poverty rate	5.5% (4.1%, 7.2%)	1.7% (0.8%, 3.5%)
		100%–185% poverty rate	7.6% (5.4%, 10.5%)	2.4% (1.3%, 4.6%)
		>185%–300% poverty rate	11.2% (7.8%, 15.7%)	1.7% (0.7%, 4.1%)
		>300% poverty rate	9.3% (7.3%, 11.8%)	2.1% (1.2%, 3.7%)
9–13 Years	Sex	Male	7.8% (6.5%, 9.2%)	2.0% (1.4%, 3.0%)
		Female	8.1% (6.8%, 9.8%)	2.6% (1.8%, 3.6%)
	Race/ethnicity	Mexican American	5.9% (4.7%, 7.3%)	1.4% (0.9%, 2.2%)
		Other Hispanic	6.5% (4.2%, 9.8%)	1.6% (0.8%, 3.4%)
		Non-Hispanic White	9.2% (7.7%, 11.0%)	2.6% (1.8%, 3.7%)
		Non-Hispanic Black	5.8% (4.4%, 7.6%)	0.9% (0.6%, 1.6%)
		Other or Multiracial	7.9% (5.3%, 11.7%)	4.5% (2.4%, 8.3%)
	Household income	<100% federal poverty rate	7.8% (6.0%, 10.1%)	1.9% (1.4%, 2.7%)
		100%–185% poverty rate	7.8% (5.6%, 10.9%)	2.3% (1.3%, 4.0%)
		>185%–300% poverty rate	7.9% (5.5%, 11.1%)	1.4% (0.6%, 3.3%)
		>300% poverty rate	8.8% (6.6%, 11.6%)	3.0% (2.0%, 4.4%)

Data presented as weighted population percent (95% confidence interval).

**Table 5:** Differences in mean *z*-scores for growth metrics by undernutrition category and age groups in US children from NHANES 2005–2014 data (*n* = 13,950).

Age Group	Measure (*z*-score)	No Indicators of Undernutrition	Mild Undernutrition	Moderate/ Severe Undernutrition	*p*
1 Year	Weight-for-length	0.60 (0.03)	−0.71 (0.06)	−1.90 (0.15)	<0.001
	Length-for-age	0.31 (0.04)	−0.22 (0.09)	−0.66 (0.18)	<0.001
	MUAC-for-age	0.06 (0.03)	−1.24 (0.04)	−1.96 (0.09)	<0.001
2–5 Years	Weight-for-height	0.56 (0.02)	−0.96 (0.02)	−2.02 (0.08)	<0.001
	BMI-for-age	0.62 (0.02)	−0.96 (0.02)	−2.06 (0.08)	<0.001
	Height-for-age	0.34 (0.03)	−0.14 (0.06)	−0.46 (0.15)	<0.001
	MUAC-for-age	0.30 (0.02)	−1.09 (0.02)	−1.60 (0.06)	<0.001
6–8 Years	Weight-for-height	0.42 (0.03)	−1.31 (0.03)	−2.18 (0.16)	<0.001
	BMI-for-age	0.64 (0.02)	−1.36 (0.02)	−2.23 (0.11)	<0.001
	Height-for-age	0.24 (0.02)	−0.20 (0.08)	−0.85 (0.22)	<0.001
9–13 Years	Weight-for-height	0.37 (0.16)	−1.09 (0.04)	−0.57 (1.48)	<0.001
	BMI-for-age	0.78 (0.02)	−1.39 (0.02)	−2.42 (0.07)	<0.001
	Height-for-age	0.42 (0.02)	−0.23 (0.07)	−0.66 (0.15)	<0.001

Data presented as mean (Standard Error). Growth metrics are significantly different across all levels of undernutrition for all measures tested with analysis of covariance, controlled for sex, race/ethnicity, and income as a percent of the federal poverty rate (*p* < 0.001).
